# IL-1 signaling enrichment in inflammatory skin disease loci with higher-risk allele frequencies in African ancestry

**DOI:** 10.21203/rs.3.rs-5724270/v1

**Published:** 2025-01-23

**Authors:** Lam Tsoi, Yumeng Dong, Matthew Patrick, Mrinal Sarkar, Haihan Zhang, Rachael Bogle, Zhaolin Zhang, Nick Dand, Michelle Paulsen, Mats Ljungman, Regina C. Betz, Lynn Petukhova, Angela Christiano, Michael Simpson, Robert Modlin, Dinesh Khanna, jonathan Barker, Irina Budunova, Mehrnaz Gharaee-Kermani, Allison Billi, James Elder, J. Michelle Kahlenberg, Johann Gudjonsson

**Affiliations:** university of michigan; University of Michigan; Department of Dermatology, INSERM 1098, Franche comté university, Besançon university hospital; King’s College London; University of Michigan; Columbia University Irving Medical Center; King’s College London; University of California Los Angeles, David Geffen School of Medicine; Department of Dermatology, University of Michigan, 1500 East Medical Center; Department of Internal Medicine, Division of Rheumatology, University of Michigan, Ann Arbor, MI, 48109, USA.; University of Michigan

**Keywords:** Genetic, Inflammatory Skin Disease, Multi-Omics, Ancestry Difference

## Abstract

Inflammatory skin diseases (ISDs) exhibit varying prevalence across different ancestry background and geographical regions. Genetic research for complex ISDs has predominantly centered on European Ancestry (EurA) populations and genetic effects on immune cell responses but generally failed to consider contributions from other cell types in skin. Here, we utilized 273 genetic signals from seven different ISDs: acne, alopecia areata (AA), atopic dermatitis (AD), psoriasis, systemic lupus erythematosus (SLE), systemic sclerosis (SSc), and vitiligo, to demonstrate enriched IL1 signaling in keratinocytes, particularly in signals with higher risk allele frequencies in the African ancestry. Using a combination of ATAC-seq, Bru-seq, and promoter capture Hi-C, we revealed potential regulatory mechanisms of the acne locus on chromosome 2q13. We further demonstrated differential responses in keratinocytes upon IL1β stimulation, including the pro-inflammatory mediators CCL5, IL36G, and CXCL8. Taken together, our findings highlight IL1 signaling in epidermal keratinocytes as a contributor to ancestry-related differences in ISDs.

## CAPSULE SUMMARY

ISD-associated loci highlight IL-1 signaling enrichment in African ancestry. Multi-omics reveals regulatory mechanisms at 2q13 for acne.

## INTRODUCTION

Inflammatory skin diseases (ISDs) encompass a group of complex genetic conditions characterized by abnormal immune responses and tissue responses ^1^. Individuals suffering from ISDs typically endure symptoms such as redness, thickening or hardening of the skin, and ISDs are associated with symptoms such as pruritus, and pain, reflecting immune cell infiltration and activation in the skin. These persistent and difficult-to-manage symptoms profoundly affect quality of life ^2^ and ISDs pose a significant public health burden. Notably, ISDs are the fourth most prevalent non-fatal condition, and are frequently associated with conditions such as allergic rhinitis, asthma, cardiovascular disease, and inflammatory bowel diseases, along with mental health issues, underscoring its extensive psychological and physiological impact ^3–9^

ISDs affect 15–20% of the global population, exhibiting marked differences in prevalence and clinical symptoms that are distinctly shaped by geographical locations and ancestry backgrounds ^3,10^. For instance, acne may be less prevalent among individuals with darker skin, yet there is preliminary evidence that patients with darker skin have elevated inflammatory responses histologically ^11^.It has been proposed that this accounts for an increased risk of post-inflammatory hyperpigmentation and keloidal or hypertrophic scarring ^12,13^. In atopic dermatitis (AD), African American children are more prone than European American children to the development of papular and follicular lesions instead of the more typical maculopapular skin lesions; thickening of the skin (lichenification) is also more common, and there may be significant hypo- or hyperpigmentation ^14,15^. In a cross-sectional study from the National Alopecia Areata Registry (NAAR), African American patients were found to have higher odds of developing alopecia areata (AA) compared to European American and Asian American patients ^16^. Among psoriasis patients, African Americans exhibit lower prevalence yet present with distinct clinical features compared to European American, including increased post-inflammatory hyperpigmentation, thicker plaques, larger areas of involvement, and less pronounced erythema ^17–19^. Structural racism may be contributing to the worsened outcomes noted among African Americans, who experience more barriers to healthcare ^20^; however, it is also known that population-specific allele frequency differences can contribute to racially associated health outcomes. In addition, some disease mechanisms are easier to de ne in populations that have an increased prevalence of disease-associated alleles. Here we seek to investigate pathogenic mechanisms behind the variations among the different ancestry groups in ISDs. Defining biological mechanisms can help improve diagnosis and treatment, reduce the healthcare burden and provide a greater level of precision medicine.

Many ISDs are complex genetic conditions, and multiple genome-wide association studies (GWAS) have been conducted to identify variants associated with ISDs ^21–30^; however, there is a notable imbalance in the ancestry composition of GWAS samples ^19,31^. An analysis from 2009 revealed that 96% of participants in GWAS were of European ancestry (EurA); by 2016, the proportion of Asian ancestry in GWAS increased, but the representation of individuals of African ancestry (AfrA), Latin American ancestry, and Indigenous backgrounds in GWAS remained almost unchanged ^32,33^. By 2022, the majority of participants in most GWAS cohorts still predominantly (> 90%) consisted of individuals of European ancestry, with only 1.5% for those of African ancestry ^34^. This underscores the need for more inclusive genetic research to understand the disparities between African ancestry and European ancestry groups that suffer from ISDs.

Skin, as the largest organ in the human body, serves as a crucial barrier and is involved in innate and adaptive immunity^35,36^. Genetic loci detected with ISD GWASs have been associated primarily with immune cells: for example, AD loci are associated with Th2 ^24,37^; and psoriasis signals enriched among enhancers in CD4 + T-helper cells (Th0, Th1, and Th17) and CD8 + cytotoxic T cells ^38^. However, keratinocytes can play crucial roles in ISDs. For instances, keratinocytes can act as active regulators of inflammation in SLE; in psoriasis, keratinocytes undergo abnormal keratinization processes like hyperkeratosis, significantly impacting the skin’s barrier function and appearance^39,40^. Furthermore, keratinocytes modulate the activity of both immune and nerve cells via the secretion of cytokines and chemokines ^36,40–43^. Therefore, exploring the regulatory mechanisms at keratinocyte loci in ISDs can enhance better understanding their role in skin inflammation, particularly given the influence of ancestry background.

The aim of this study is to leverage ancestrally derived differences in allele frequencies among ISD-associated loci to identify diseases-shared pathway contributing to differential inflammatory responses in keratinocytes among AfrA.

## RESULTS

### ISDs-Associated Signals

**Figure 1:**
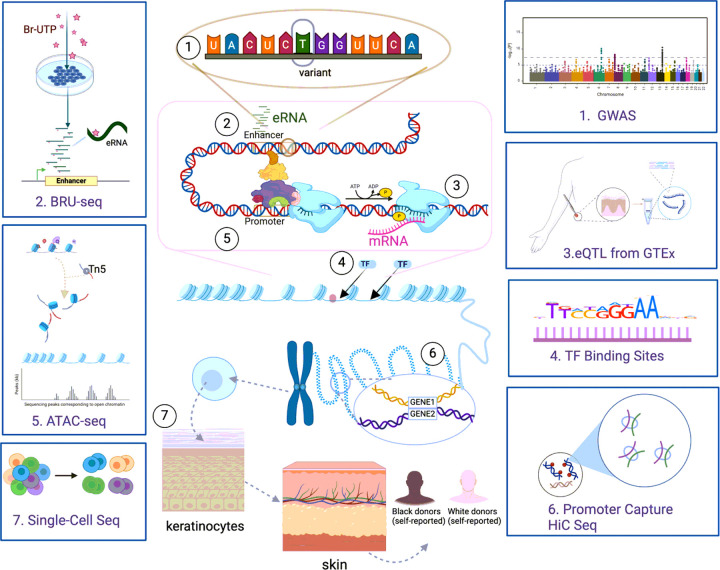
Study overview. We collected GWAS loci from seven ISDs and computed the BCI variants for each locus. We utilized multi-omic data to ne-map the potential causal variants and reveal the regulatory mechanism: Bru-seq was used to detect eRNAs in keratinocytes; skin eQTL data from GTEx to reveal association between genotypes and expression profiles; ENCODE ChIP-seq data to identify TF binding sites; ATAC-seq assessed chromatin accessibility in keratinocytes; pc-HiC seq revealed physical interactions within the 3D chromosomal structure of keratinocytes; and single-cell sequencing displayed gene expression in keratinocytes. This figure is created with BioRender.com.

**Figure 2:**
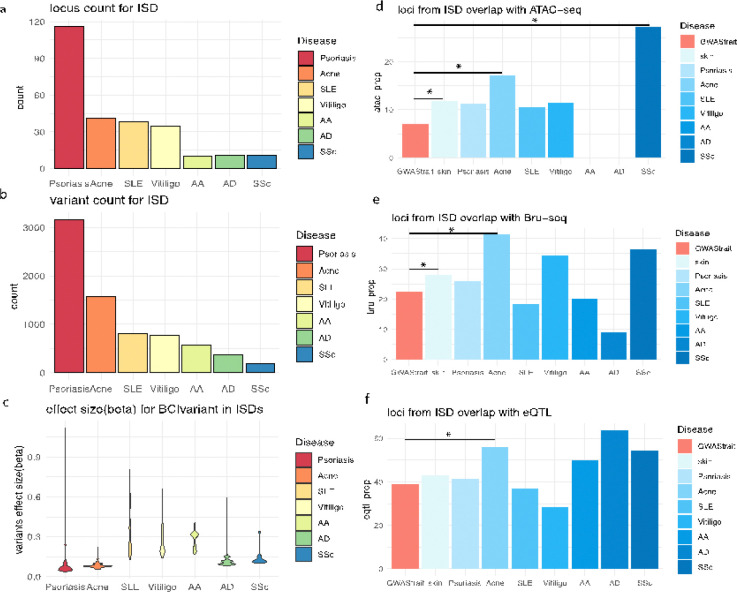
GWAS signals for ISD. (a) GWAS loci count for each ISD; (b) BCI variant count for each ISD; (c) risk allele effect sizes (beta) of the BCI variant demonstrate variability across different ISDs; (d) the proportion of ISD loci that overlap with ATAC peaks, compared with other GWAS traits (red); (e) the proportion of ISD loci that overlap with the Bru-seq signal, compared with other GWAS traits; (f) the proportion of ISD loci that overlap with the eQTL signal, compared with other GWAS traits. (binomial test, p-value<0.05 marked as *)

Figure 1 provides a schematic overview of the analytical workflow. We collected GWAS summary statistics from seven common ISDs: Acne, AA, AD, Psoriasis, SLE, SSc, and Vitiligo. The number of disease-associated loci varied from 10 (SSc) to 116 (Psoriasis) (Fig 2A; **see** Methods). We identified potential causal variants by computing the 95% Bayesian Credible Intervals (BCI) for each disease-associated signal, resulting in 7,454 potential causal variants from 273 disease signals, with the number of BCI variants for each disease varying from 197 (SSc) to 3,156 (psoriasis) (Fig 2B). Notable differences were observed in the effect size distribution of the risk allele for the BCIs (Fig 2C): psoriasis and acne have the largest numbers of disease signals, but they also exhibit, on average, the lowest effect sizes, predominantly below 0.15 (ORs=1.2). While the observations can be attributed to the power / sample size (see Methods) / prevalence of the individual ISD GWAS, the findings can also indicate that these ISDs have different complexity in regulatory mechanisms.

To evaluate the regulatory roles of these ISD signals in keratinocytes, we first utilized ATAC-seq data from keratinocytes of healthy skin donors to ascertain chromatin accessibility. We observed the ISD signals to be significantly overlapping with the peaks identified by the ATAC-seq data (binomial test, Obs/Ex(OE)=1.67; *p=5.3′*) (Fig 2D) when comparing against the overlap with other complex disease loci from the GWAS catalog ^44^, with acne (OE=2.41; *p=2.4′10*^*−2*^) and SSc (OE=3.84; *p=3.8´10*^*−2*^) showing the most notable overlaps. Active enhancers can be transcribed as enhancer RNA (eRNA)^45^, and we employed Bromouridine sequencing (Bru-seq) to profile the eRNA in keratinocytes. Coincidently, we observed significant overlap between the ISDs signals and regulatory regions captured by Bru-seq from N/Tert cell line, when comparing to GWAS loci from other complex traits (OE=1.25; *p=3.8′10*^*−2*^), with acne showing particularly marked significance (OE=1.85; *p=7.0′10*^*−3*^) (Fig 2E). Indeed, acne signals also have significant overlap with skin eQTLs derived from GTEx (OE=1.44; *p=3.6′10*^*−2*^). We observed most of the ISDs to have signals overlapping with eQTLs to a higher degree than those from other complex traits (Fig 2F; **see** Methods), though they are not statistically significant. We then investigated the specificity of ISD signals in keratinocytes, by utilizing ENCODE data encompassing a broad range of cell types. ISD signals overlapping with our ATAC peaks with on average 2.6 fold more overlap with POL2 binding site in NHEK, when comparing with that from other cell types. The above multi-omic results highlight the pivotal regulatory roles played by the ISD signals in keratinocytes.

### ISD Loci with higher RAF in AfrA Indicate IL1 Signaling In ISD

**Figure 3:**
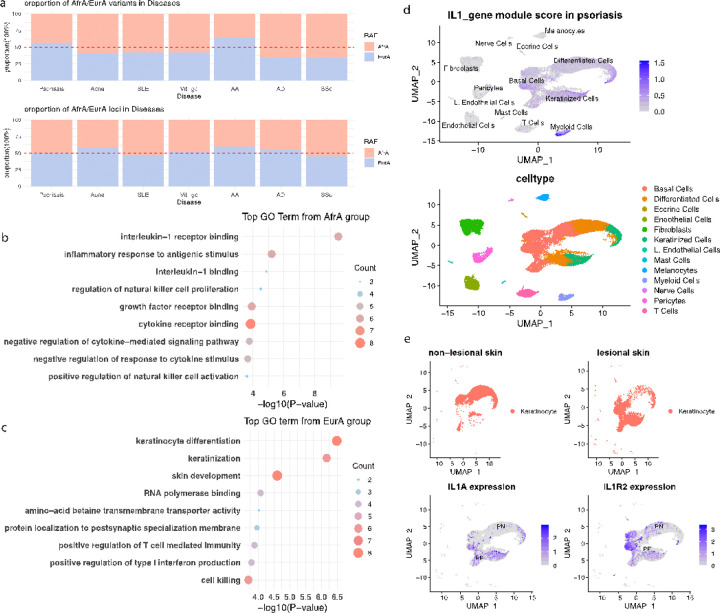
IL-1 signaling among ISD signals with higher RAFs in AfrA (a) the proportions of BCI variants (top) or loci (bottom) for ISDs having higher RAF in AfrA/EurA; (b) enrichment analysis result for eQTL targeted genes of the ISDs signals with RAFs higher in AfrA (b) or EurA (c); (d) Module score computed using eQTL targeted genes exhibit elevated expressions in keratinocytes (in addition to myeloid) comparing against other cell types ; (e) Up-regulation of IL1A and IL1R2 in lesional skin compared to non-lesional skin in psoriasis scRNA.

We then studied population RAF of the ISDs GWAS signals in AfrA and EurA. While there was no significant difference between the RAFs between AfrA and EurA at the ISD loci lead variant level, for 5 of the 7 diseases, we observed higher RAFs for BCI variants in AfrA (when compared to EurA), with the contrast being the highest among SSc (66.0% in AfrA) and AD (65.0% in AfrA) (Fig 3A).

We then jointly modeled if higher AfrA RAF in BCI variants is associated with different chromatin/genomic marks in keratinocytes, including ATAC peaks, Hi-C loop ends, Bru-seq signals, as well as eQTL status in skin. Our results illustrate that higher AfrA RAF is significantly associated with skin eQTL (OR=1.4, *p = 3.6′10*^*−12*^) and the presence of eRNA (OR=1.3, *p = 7.1′10*^*−7*^). When testing against each chromatin mark individually, in addition to Bru-seq signal (OR=1.3, *p=2.7′10*^*−8*^) and skin eQTL (OR=1.4, *p =7.4′10*^*−12*^), we also revealed that promoter capture Hi-C loop is enriched among BCI variants with higher AfrA RAF (OR=1.2, *p=5.8′10*^*−3*^). These results suggest that the ISD-associated variants that are more prevalent among AfrA individuals tend to play regulatory roles in keratinocytes; however, we did not identify significance when extending to locus level.

To elucidate the functional pathways associated with these observations, we conducted enrichment analyses on the eQTL-targeted genes of the ISD loci. Our analysis identified that the loci with higher AfrA RAF are enriched for interleukin 1 receptor binding (OR=103.4, *p=3.03′10*^*−10*^), positive regulation of natural killer cell proliferation (OR=81.2, *p=1.8′10*^*−5*^), and positive regulation of leukocyte activation (OR=5.4, *p=4.0′10*^*−5*^) (Fig 3B). In contrast, the ISD loci with higher EurA RAF exhibit enrichment in pathways related to keratinocyte differentiation (OR=14.0, *p=3.3′10*^*−7*^) and skin development (OR=7.6, *p=2.5′10*^*−5*^) (Fig 3C).

Specifically, eQTL targets of the ISD signals with higher AfrA RAFs involved in the interleukin 1 signaling pathway include *IL1A, IL1B, IL1RN, IL1R2, IL36B, IL36RN*, and *IL37*. We investigated the expression profiles of these genes in a large scRNA cohort of psoriasis skin ^46^. A module score computed using these genes exhibit elevated expressions in keratinocytes (in addition to myeloid cells) when comparing against other cell types (Fig 3D). Individually, we demonstrated that *IL1A* (FC=1.05, *p=5.9′10*^*−70*^) and *IL1R2* (FC=1.1, *p=4.5′10*^*−64*^) were up-regulated in the lesional skin compared to non-lesional skin (Fig 3E). To reveal the potential upstream regulators, we then utilized ChIP-seq data from ENCODE ^47^ to identify transcription factors (TFs) binding to the IL1 signaling loci with higher AfrA RAFs. We highlighted multiple TFs with binding sites overlapping with these loci, including FOXA1, Estrogen receptor (ER), as well as GATA1. A previous study also illustrated GATA1 binding to *IL1A* promoter using chromatin immunoprecipitation assays ^48^.

### Regulatory Mechanisms Of IL1 Signaling

**Figure 4:**
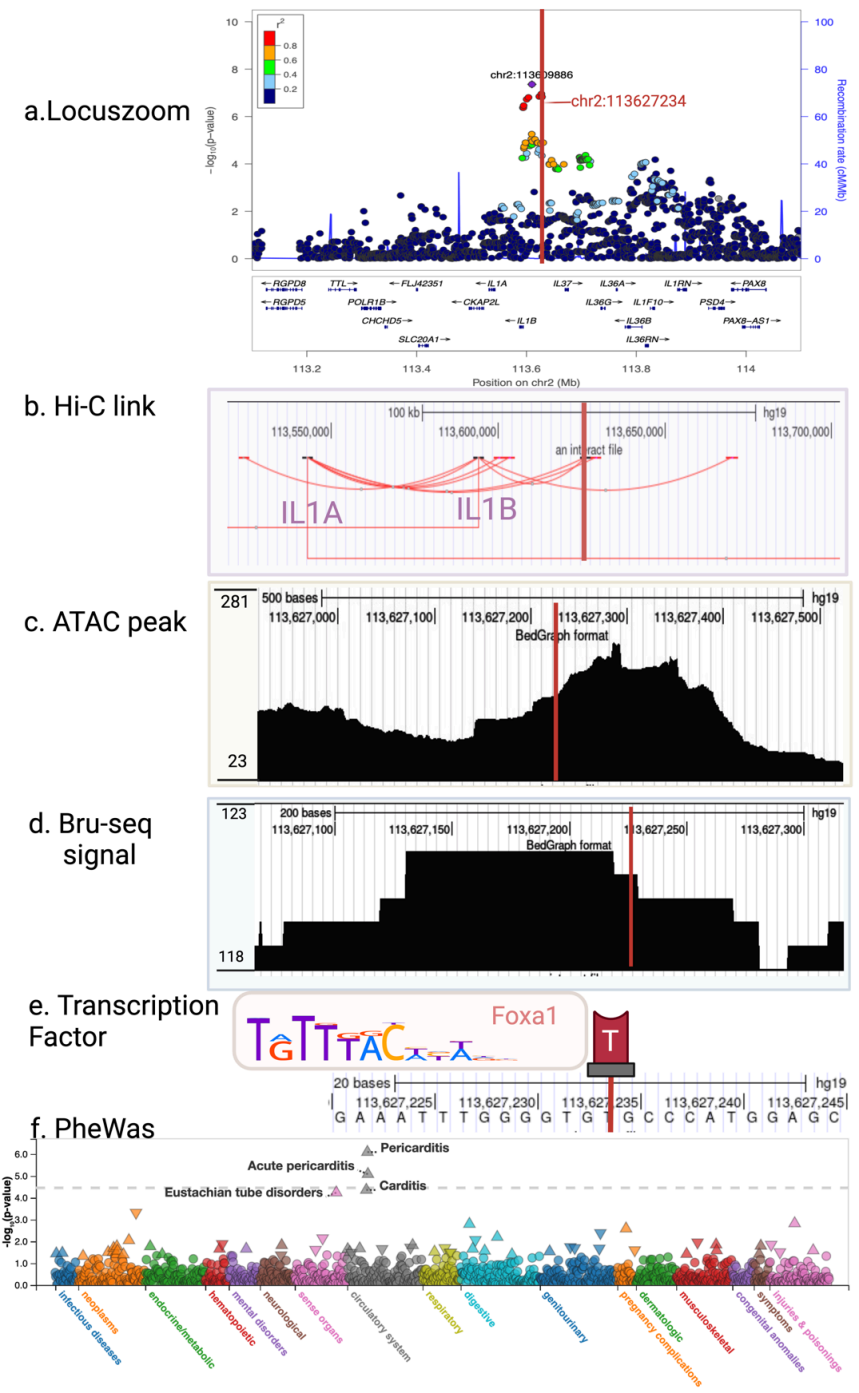
Multi-omic information to ne-map 2q14.1. (a) regional association plot of acne locus 2:113609886_C_T (2q14.1), with the red line marks the BCI variant chr2:113627234_T; (b) we performed promoter capture Hi-C in keratinocytes to reveal interactions between region encompassing BCI variant chr2:113627234_T and the promoter regions of *IL1A* and *IL1B*, complementing the eQTL results; (c) ATAC peaks reveals that the variant is located within an open chromatin region in cultured human primary keratinocytes, indicative of a zone of active gene regulation; (d) Bru-seq reads was detected covering this region, suggesting transcribed active enhancer element; (e) ChIP-seq from keratinocytes have identified multiple transcription factors peaks that bind to this variant, FOXA1 as illustrated; (f) from PheWas, we discovered a significant association between the variant chr2:113627234:T and pericarditis(effect size 0.21, p-value of 8.1′10^−7^) or acute pericarditis(effect size 0.46, p-value of 8.3′10^−6^) from circulatory system. This figure is created with BioRender.com.

Table 1 summarizes our findings for the ISD-associated loci encompassing the IL1 pathway genes. Using this information, we integrated evidence from variant mutations to transcriptional impacts, and further explored the interaction between promoters and enhancers, to understand the differences between AfrA and EurA. As a proof of concept, we use the acne-associated signal on chromosome 2q13 in keratinocytes as an example (Fig 4A) ^49^. This locus encompasses nine BCI variants, but only rs1143627 and rs2708914 are supported by comprehensive omic data for their regulatory roles in keratinocytes. First, we identified loops in promoter capture Hi-C (pc Hi-C) in keratinocytes encompassing the acne-associated variants, highlighting that both variants interact with the promoter region of *IL1A*, with rs2708914 also connected to *IL1B*, complementing the eQTL results (Fig 4B). Specifically, rs2708914 is associated with five eQTL target genes (*IL1A, IL1B, IL1RN, IL36B*, and *IL37*). It is located within an open chromatin region captured by ATAC-seq peaks in keratinocytes, indicative of a zone of active gene regulation (Fig 4C). This is further supported by Bru-Seq data, indicating nascent eRNAs were expressed in this region (Fig 4D). In addition, ChIP-seq experiments have identified multiple transcription factor peaks, including AP2-, AP2-, STAT1, STAT3, as well as FOXA1, which has binding sites overlapping with two loci (2q13 for acne and 2q11.2 for psoriasis) (Fig 4E). Notably, we found that rs2708914 is in the middle of the putative FOXA1 binding motif assayed by ChIP-seq, and it is one of the most evolutionary constrained positions in the region based on genomic evolutionary rate profiling (GERP) score based on multi-species sequence alignment. Together, these results delineate a detailed map of genetic regulation, demonstrating how rs2708914 influences gene expression in keratinocytes through changes in chromatin state, enhancer activity, and transcription factor binding, ultimately impacting the pathophysiology of ISDs. Our data further provides insight to reveal the complex network of transcriptional regulation changes associated with the IL1 pathway in ISDs.

We also studied the clinical implications of this genetic signal beyond complex immune skin conditions. The PheWAS results from TOPMed-imputed UK Biobank ^50–52^ highlights the significant association between the variant rs2708914 and pericarditis (OR=1.23, *p=8.1′10*^*−7*^) and acute pericarditis(OR=1.58, *p=8.3′10*^*−6*^) (Fig 4F). Notably, a recent GWAS study on pericarditis revealed a variant (rs12992780, with risk allele T) near *IL1B* ^53^. Linkage disequilibrium (LD) analysis between rs12992780 and rs2708914 revealed that the theoretical frequency of risk allele T-T haplotypes was 0.114, while the observed frequency was 0.236. An LD-r² value of 0.3 and a p-value of < 0.001 indicates a significant correlation between the two variants. This finding underscores the potential systemic impact of variant rs2708914.

**Figure 5:**
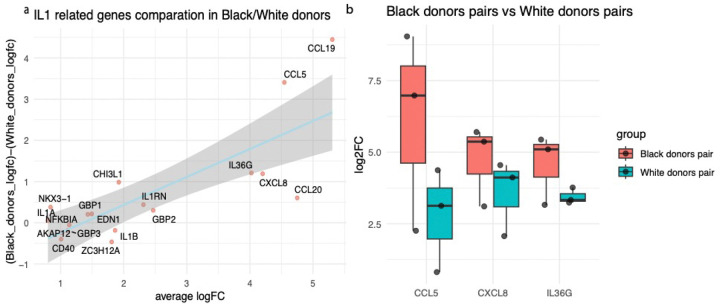
IL1 stimulation shows elevated effect in AfrA keratinocytes (a) the difference of fold change of IL1-stimulation between Black donors (self-reported) and White donors (self-reported) keratinocytes (y-axis) versus the average IL1-stimulation fold change (all genes with FC>1.5 and p-value<0.05 are being shown); (b) the individual sample fold changes for *CCL5, CXCL8*, and *IL36G*in Black donors and White donors.

To further validate the elevated IL1 signaling in AfrA keratinocytes, we isolated keratinocytes from skin biopsies from individuals self-reported as African ancestry or European ancestry (see Methods), to understand the genome-wide expression differences in IL1 stimulation effect through bulk RNA-seq. Interestingly, among the IL1 induced genes (FC>1.5, FDR<=10%), we observed that the IL1 response between AfrA and EurA to be positively correlated with the average degree of IL1 response (Fig 5A; Pearson’s correlation=0.81, *p=7.0′10*^*−4*^). Some of the most prominent examples include *CCL5, CXCL8*, and *IL36G*, with higher average IL1 responses observed in AfrA (Fig 5B). This result further corroborates the differences in IL-1 signaling between AfrA and EurA.

## DISCUSSION

Our study provides a comprehensive overview of the genetic differences between AfrA and EurA for ISD-associated loci, specifically highlighting the IL-1 signaling pathway among loci that have greater allele frequencies among AfrA. This analysis delineates the regulatory mechanisms of a key variant for 2q13, as revealed by multi-omics data. These findings have identified a mechanism that had greater statistical power to be detected among AfrA individuals. It remains to be determined how this mechanism is contributing to variations in skin inflammation between different ancestry groups. It also paves the way for more precise, tailored therapeutic approaches.

ISDs exhibit notable variability across different ancestry background group. AfrA individuals have been observed to have a higher incidence of certain conditions such as SLE ^54,55^, and AD ^56,57^, than their EurA counterparts. Despite lower incidence of psoriasis, AfrA patients have unique disease manifestation and potentially higher severity, drawing attention to differences in underlying causes ^19^. Studies indicate that differences in skin structure (dark skin has more corneocyte layers within similar thickness) ^58,59^, social-psychological factors (access to healthcare) and cultural factors (e.g. white women are more likely to use makeup to cover acne than non-white) ^13,60–62^ may contribute to these disparities. Recent GWAS have underscored the genetic factors influencing these ISDs; however, the predominance of European ancestry cohorts limit their generalizability ^26,31,34^. Our study compared the RAFs of genetic loci for different skin diseases between AfrA and EurA populations to highlight existing disparities.

Our results suggest the impact of IL1 signaling in ISDs and identified IL1 signaling as a potential factor differentiating the inflammatory manifestations between AfrA and EurA. The IL1 family is a growing group of cytokines with both pro-and anti-inflammatory activities, including the agonists IL1α, IL1β, IL18, IL33, IL36 α, IL36 β and IL36 γ, the antagonists IL1Ra, IL36Ra and IL38, and the anti-inflammatory cytokine IL37 ^63,64^. They are essential in the regulation of pro-inflammatory responses within the innate immune system, with IL1α and IL1β being the primary effectors ^65–67^. IL1α secreted by keratinocytes can stimulate neutrophils, activates itself, and therefore act as an inflammatory response amplifier. While IL1α influences the local micro-environment, IL1β acts as a regulator of systemic inflammation, which is well-known in ISDs ^68–70^. IL1Ra (encoded by *IL1RN*) engages the binding site of ILR1 without recruiting the co-receptor IL1R3, thus blocking IL1α and IL1β signal transmission ^64,71^. It is widely believed that the ratio of IL1α or IL1β to IL1Ra is a contributing or determining factor in inflammatory diseases, such as polymorphic light eruption, psoriasis and AD ^64,72,73^. The IL36 family, including IL36α, IL36β, IL36γ, is also involved in IL1 signaling ^74^.

Focusing on the inflammatory signaling differences across ancestral groups allows us to leverage allele frequency differences that can enhance detection of some genetic mechanisms. It also sets a foundation for future studies to better the differences in disease prevalence and clinical disparities between different ancestry groups. Previous studies have demonstrated that 3D human skin equivalents derived from primary keratinocytes of AfrA individuals are more responsive to the pro-inflammatory effects of TNFα compared to those from EurA, potentially explaining disease outcomes that are associated uniquely with the development of ISDs in AfrA populations ^75^. Our study highlights a difference in IL1 signaling in ISDs among the AfrA population, underscoring the need for further research across a more diverse demographic and at the protein level. Our results also hint that the acne variant rs2708914 can play an additional pathological role in other conditions, including pericarditis. Previous studies showed that IL1β can be activated by the NLRP3 inflammasome, which plays a central role in acute pericarditis clinical manifestations. Anakinra, a recombinant IL1Ra, and IL1 inhibition with rilonacept are also used for pericarditis treatment ^76,77^.

We propose some insights into how variants associated with IL1 signaling may contribute to the mechanisms of skin diseases in ancestry differences. such as the acne locus 2q13. We believe these insights can provide more comprehensive evidence and help narrow down the scope of screening, and this work serves as a bridge connecting large-scale genomics data with future experimental validation such as CRISPR-based method.

Our study provides evidence from multiple levels, ranging from BCI variant to epigenetic modifications to the transcriptome, strongly supporting that the acne locus 2q13 contributes to heightened IL1 function in keratinocytes in the AfrA population. However, further validation in a larger population is necessary, and we hope that future studies can employ CRISPR-Cas9 for experimental verification. Our results identify a disease mechanism contributing to clinical features that vary among different ancestry groups and highlights IL1 responses as a central pathway in skin inflammation.

## METHODS

### GWAS summary statistics

The overview of our work is laid out in Fig. 1. We retrieved the GWAS summary statistics for 7 ISDs: Acne (20,165 cases and 595,231 controls) ^49^, AA (2,332 cases and 5,233 controls) ^78^, AD (13,287 cases and 41,345 controls) ^79^, Psoriasis (11,024 cases and 16,336 controls) ^23^, SLE (7,219 cases and 15,991 controls) ^80^, Systemic Sclerosis (9,095 cases and 17,584 controls) ^29^ and vitiligo (2,853 cases and 37,405 controls) ^28^. We selected single nucleotide polymorphisms (SNPs) with p-values below the genome-wide significance threshold of 5×10^− 8^. Since we used summary statistics of the GWAS in this work, we excluded MHC in our analysis due to its complexity nature of the linkage disequilibrium. Non-MHC loci were delineated based on distance ( > = 500kb) for each disease and 95% Bayesian credible sets were computed. In total, we identified 7,454 variants from 262 loci for the 7 ISDs. The positions of the loci and variants we utilized are derived from GRCh37/hg19. For each disease-associated variant, we acquired risk allele frequencies (RAF) for AfrA, including Yoruba in Ibadan, Nigeria(YRI), Luhya in Webuye, Kenya(LWK), Gambian in Western Division, The Gambia(GWD), Mende in Sierra Leone(MSL), and Esan in Nigeria(ESN)) and EurA (including Utah residents with Northern and Western European ancestry(CEU), Toscani in Italy(TSI), British in England and Scotland(GBR), Finnish in Finland(FIN), and Iberian populations in Spain(IBS) from the 1000 Genomes Project Phase 3 ^81^ using Plink 2 ^82^. We also obtained disease/trait-associated variants for 11,860 traits from the GWAS catalog “GWAStrait” ^44^ for comparison in our analysis. Variants within 500kb range were considered in the same locus, and the one with the smallest p-value was selected as the best signal ^44^.

### Multiome data to understand regulatory roles in keratinocytes

We integrated skin eQTL targeted data from sun-exposed and non-sun-exposed skin tissue data from the Genotype-Tissue Expression (GTEx) project ^83^, using variant-gene pairs with nominally significant (p < 0.05) p-values. We conducted ATAC-seq (Assay for Transposase-Accessible Chromatin with high-throughput sequencing) using primary keratinocytes from 4mm healthy individuals’ skin biopsies. Biopsies were incubated in 0.4% Dispase II (Gibco, Thermo Fisher Scientific, cat #17105041) overnight to separate the epidermis and dermis. After the separation, the epidermis was transferred to 0.25% Trypsin-EDTA + 10 units/mL DNase mixture and incubated at 37°C for 1hr. The Epidermis mixture was then quenched with FBS and precipitated by centrifugation. Cell pellets were then resuspended in PBS + 0.04% BSA. Cell numbers were counted at this step for future dilution calculation. Cells were harvested and subjected to transposition using the Tn5 transposase, which fragments DNA and simultaneously inserts sequencing adapters into accessible chromatin regions. Following transposition, DNA was puri ed and then amplified via PCR to enrich tagged fragments and incorporate sequencing indices. The amplified library was puri ed, quantified, and then underwent sequencing.

We conducted triplicates for the Bromouridine sequencing (Bru-Seq) experiments. N/TERT-2G cells ^84^ were grown on a 150 mm cell culture plate until they reached 80–90% confluency, containing approximately 5–6 million cells, with each plate containing 10 ml in KSFM (Keratinocyte Serum-Free Medium, Gibco, 0.09mM [Ca2+], cat #17005042) with 1M [Ca2+] adjusted to 0.4 mM. BrU (Bromouridine; Millipore-Sigma #850187) was added to cell culture media to obtain final concentration of 2 mM before incubating at 37°C for 30 minutes. We then added 3 ml of TRIzol (Invitrogen #15596026) to the plate, and cells were collected using a cell scraper and transferred into a 15 ml tube. The enriched BrU-labeled RNA was then converted into a cDNA library for sequencing.

Promoter capture HiC seq (pc-HiC seq) was also performed from N/TERT 2G ^84^, which was cultured in KSFM (Gibco, 0.09mM [Ca^2+^], cat #17005042) with 1M [Ca^2+^] adjusted to 0.4 mM. We let cells grow for 3 days, at which the cells are confluent or near-confluent, then let the cell grow for another 3–4 days. We prepared the Capture-HiC libraries using the Arima Capture-HiC Kit (Arima-HiC + for promoter capture HiC) according to the manufacturer’s protocol (Arima genomics).

For IL1β stimulated keratinocytes, punch biopsies were obtained from University of Michigan patients enrolled in the Taubman Institute Innovative Program PerMIPA cohort, including two patients diagnosed with SLE and one healthy control who self-identified as African ancestry were selected for this study. Correspondingly, two SLE patients and one healthy control who self-identified as European ancestry were also included. We obtained 6-mm punch biopsies from these participants. The epidermis was separated from the dermis after overnight in 0.4% dispase II (Gibco, Thermo Fisher Scientific, cat #17105041). Isolated keratinocytes were cultured in KSFM (Gibco, 0.09mM [Ca^2+^], cat #17005042) with HKGS (Human Keratinocyte Growth Supplement, Gibco, cat #S0015) at 37°C in a 5% CO₂ environment. Keratinocytes were divided into two groups: one treated with 10ng/ml IL1β (R&D systems, cat #201-LB-005) for 6 hours and one untreated control. Both groups were assessed post-treatment to analyze IL1β effects.

### Data processing

ATAC-seq After adapter trimming, we conducted BWA alignment to map reads to human reference genome(hg19) ^85^. Only uniquely mapped reads were used; mitochondrial reads and one of the read duplicates were removed. ATAC-seq peaks were computed using MACS2 ^86,87^.

Bru-Seq We mapped reads to the human genome, and we established the top 25% signal threshold as the criterion for significant Bru-Seq readings, with thresholds defined against the respective background.

pc-HiC seq We analyzed using the software CHiCAGO to identify loops ^88^. To be specific, from the (pre-designed) promoter regions of interest, we identified chromosomal loops where the other ends were captured by the baits (the captured end). These were different from regular HiC loop profiles, since the bait end of a pc-HiC loop was pre-designed, and only the captured end of a pc-HiC loop was overlapped with known ISDs loci. The number of disease-overlapped captured ends were calculated.

Bulk RNA-seq After adapter trimming, we conducted alignment to map reads to human reference genome (hg19) using STAR ^89^. Only uniquely mapped reads were used in the analysis. Gene quanti cation was performed using HTSseq ^90^.

### Downstream analysis

We utilized Generalized Linear Models (GLMs) to assess the association between elevated RAF in AfrA individuals (AfrA = 1; EurA = 0) and other variables including eQTL status, as well as overlap against ATAC-seq peaks, pc-HiC interaction loops, and enhancers captured by Bru-Seq. The significance of the predictors was determined by the estimated coefficients and their associated P values.

We performed function enrichment from the GO (Gene Ontology) dataset ^91^ using the R packages ‘org.Hs.eg.db ^92^‘ and ‘GOstats ^93^‘. Use GO ontologies (‘BP’, ‘CC’, ‘MF’), and filter for term sizes less than 500. We analyzed the data with the R package ‘seurat ^94^‘, ‘tidyverse ^95^‘,’gridextra ^96^‘,’limma ^97^‘,’DESeq2 ^98^‘. To obtain comprehensive genes for IL1 signaling, AmiGO2 ^99^ was used for retrieving genes involved in the IL1 signaling pathways, thus including: IL1 receptor family member, IL1 family member, IL1 receptor-associated kinase, IL1 receptor-associated kinase 1-binding protein 1, Celluar response to IL-1, and IL1 receptor binding. We obtained 154 unique genes in total.

LDlink between variants was performed by LDpair Tool ^100^, with Genome Build in GRCh37, using samples from African (AFR) and European (EUR) groups. The cross-disease variant association was conducted by PheWeb using PheWAS (Phenome-Wide Association Study) data from UK Biobank ^50^ and TOPMed-imputed ^51,52^.

## Figures and Tables

**Table 1.** IL1 related loci with multi-omic evidence

Disease	Locus (Hg19)	BCIvariant: RA	AfrA RAF	EurA RAF	eQTL.GTEx_Skin Targeted Genes (Hi-C Targets Are Bolded; The Underscore Indicates The IL1 Related Gene).	ATAC-Seq Peaks	Bru-Seq Signal	Overlap With Transcritpion Factor Peaks
Acne	2q13	2:113628301:C	**0.63**	0.34	*CDK8P2, **IL1A**, IL1B, IL1RN, IL36B, IL37*		YES	
		2:113627234:T	**0.63**	0.34	*CDK8P2, **IL1A**, **IL1B**, IL1RN, IL36B, IL37*	YES	YES	FOXA1, POL2, TCF4, KAP1, AP2ALPHA, AP2GAMMA, CEBPB, CJUN, JUND, MXI1, P300, STAT1, GATA2, CMYC, EJUNB, EJUND, STAT3, GATA3
		2:113594387:G	**0.57**	0.35	*CDK8P2, **IL1A**, IL1RN, IL36B, **IL37***	YES	YES	POL2, PU1, CEBPB, E2F6, MAX
		2:113594867:A	**0.57**	0.35	*CDK8P2, **IL1A**, IL1RN, IL36B, IL36RN, **IL37***	YES	YES	
AA	11q 13.1	11:64053157:T	**0.97**	0.63	*CCDC88B, PLCB3, PPP1R14B, PRDX5, RPS6KA4*	YES		POL2, ERALPHAA, ELF1, NFKB, SETDB1, FOSL1, GTF2B, HMGN3, SIX5, THAP1
		11:64052447:C	**0.94**	0.63	*CCDC88B, FERMT3, **PLCB3**, **PPP1R143**, PRDX5, **RPS6KA4***	YES		POL2, NFKB, TAF1, TBP, HEY1, HAE2F1
		11:64102948:A	**0.97**	0.62	*CCDC88B, PPP1R14B, PRDX5, RPS6KA4*	YES		EGR1, POL2, E2F6, CTCF, ELF1, GATA1
AD	2q 12.1	2:103060024:T	0.10	**0.22**	*IL18R1*	YES		CTCF
Psoriasis	2q11.2	2:102589726:A	0.37	**0.67**	*IL1R2, LINC01127*	YES		STAT3,CMYC
		2:102600083:C	**0.94**	0.69	*IL1R2, LINC01127*	YES		ERALPHAA,FOXA1
		2:102622818:A	**0.95**	0.70	*IL1R2, LINC01127*	YES		CTCF,RAD21,SMC3,ZNF143
	5q15	5:96118852:G	0.29	0.29	*CTD-2260A17.1, CTD-2260A17.3, ERAP1, ERAP2*		YES	
	11q13.1	11:64052447:C	**0.94**	0.63	*CCDC88B, FERMT3, **PLCB3**, **PPP1R14**, PRDX5, RPS6KA4*	YES		POL2,NFKB,TAF1,TBP,HEY1,HAE2F1
		11:64053157:T	**0.97**	0.63	*CCDC88B, PLCB3, PPP1R14B, PRDX5, RPS6KA4*	YES		POL2,ERALPHAA,ELF1,NFKB,SETDB1,FOSL1,GTF2B,HMGN3,SIX5,THAP1
		11:64102948:A	**0.97**	0.62	*CCDC88B, PPP1R14B, PRDX5, RPS6KA4*	YES		EGR1,POL2,E2F6,CTCF,ELF1,GATA1
SLE	2q13	2:113829869:G	**0.91**	0.72	***IL1A**, IL36B, IL36RN*	YES		

eQTL p-values<1’10^−5^ besides 2:113829869-IL36RN(p=5.7’10^−3^), 11:64052447-PLCB3(p=3.0’10^−2^), 11:64052447-RPS6KA4(p=5.0’10^−5^)

## Abbreviations

AA: Alopecia Areata
AD: Atopic Dermatits
AfrA: African Ancestry
ATAC-seq: Assay for Transposase-Accessible Chromatin with high-throughput sequencing
BCI: Bayesian Credible Intervals (BCI)
Bru-seq: Bromouridine Sequencing
Bru: Bromouridine
eQTL: Expression Quantitative Trait Loci
eRNA: enhancer RNA
EurA: European Ancestry
GERP: genomic evolutionary rate profiling
GLMs: Generalized Linear Models
GO: Gene Ontology
GTEx: Genotype-Tissue Expression
GWAS: Genome-Wide Association Studies
ISDs: Inflammatory Skin Diseases
KSFM: Keratinocyte Serum-Free Medium
LD: Linkage disequilibrium
pc-HiC seq: Promoter capture HiC seq
PheWAS: Phenome-Wide Association Study
RAF: Risk Allele Frequency
SLE: Systemic Lupus Erythematosus
SNPs: Single nucleotide polymorphisms
SSc: Systemic Sclerosis
